# Discovery and Field Evaluation of Sex Pheromone Components for the Click Beetle *Melanotus verberans* (LeConte) (Coleoptera: Elateridae)

**DOI:** 10.1007/s10886-025-01569-3

**Published:** 2025-02-05

**Authors:** Livy Williams III, Sean T. Halloran, Paul D. Baker, Frank E. Etzler, Lance L. Lawrence, Jocelyn G. Millar

**Affiliations:** 1 USDA-ARS U.S. Vegetable Laboratory, 2700 Savannah Highway, Charleston, SC 29414 USA; 2 Departments of Entomology and Chemistry, University of California, Riverside, CA 92521 USA; 3 Department of Plant and Environmental Sciences, Clemson University Pee Dee Research and Education Center, 2200 E. Pocket Road, Florence, SC 29506 USA; 4 Montana Department of Agriculture, 302 N. Roberts, Helena, MT 59714 USA

**Keywords:** Elateridae, Click beetles, Wireworms, *Melanotus verberans*, Sex pheromone, Attractant, Sustainable pest management

## Abstract

Wireworms, the larval stage of elaterid beetles, are among the most serious soil-borne insect pests in the world. Wireworms feed on a variety of key crops, including small grains, maize, vegetables, fruits, sugar cane, sugar beets, potatoes, and sweet potatoes. Management of these pests is becoming increasingly problematic, in part due to knowledge gaps in their basic biology, which hinders development of effective crop protection strategies. In particular, little is known about the semiochemicals that mediate the reproductive behavior of these pests. Research over the past two decades has begun to fill this need, with: (1) the discovery of sex attractants for several key pest species, and (2) subsequent studies toward development of semiochemically-based pest management approaches. We used chemical and behavioral studies to identify, synthesize, and field test the sex attractant pheromone of adult *Melanotus verberans*, the larvae of which are important crop pests. In coupled gas chromatography-mass spectrometry analyses of extracts of ovipositors of females, five possible pheromone components were identified. Subsequent coupled gas chromatography-electroantennogram detection analyses indicated that male antennae were responsive to only two of these compounds, 13-tetradecenyl acetate and 13-tetradecenyl hexanoate. In field trials, neither compound alone was attractive, but a blend of the two was strongly attractive to conspecific male beetles, and did not attract other species. A two-year field study showed that most male beetle flight activity occurred from April through May. Because the sex pheromone of *M. verberans* consists of two compounds that can be readily synthesized, its development for integrated pest management should be economically feasible.

**Supplementary Information:**

The online version contains supplementary material available at 10.1007/s10886-025-01569-3.

## Introduction

The North American click beetle fauna (Coleoptera: Elateridae) include nearly 1,000 described species (Johnson 2002). Many are valuable members of natural ecosystems, where they recycle biomass by feeding on detritus, or are predatory. However, some are important pests of a wide array of crops (reviewed in Rashed and van Herk 2024; Traugott et al. 2015). Regardless of their pest status, little is known about the biology and natural history of most species, in part due to the concealed habitats of immature stages (i.e., subterranean, decaying wood, litter), and the cryptic appearance of adults of most species (Douglas et al. 2024). Furthermore, many species have long life cycles of several years, making them difficult and expensive to rear and study.

The reproductive biology of click beetles has been a growing focus of research over the past ~ 20 years, and the available evidence indicates that females of many species produce sex attractant pheromones to attract males for mating. For example, pheromones and sex attractants have been identified for a number of European and Asian species (e.g., Tamaki et al. 1990; Tolasch et al. 2007; reviewed in Tóth 2013). The first pheromones for North American species were only reported in 2018, for two *Cardiophorus* Eschscholtz species (Serrano et al. 2018). Since then, pheromones or sex attractants have been discovered for several pest species, including *Melanotus communis* (Gyllenhal) (Williams et al. 2019), *Limonius californicus* (Mannerheim), *L. canus* LeConte (Gries et al. 2021), L. *agonus* (Say), and *L. infuscatus* (Motschulsky) (van Herk et al. 2021a), and *Selatosomus destructor* (Brown) (Gries et al. 2022). More recently, pheromones or sex attractants have also been reported for *Parallelostethus attenuatus* (Say) (Millar et al. 2022), *Idolus* Desbrochers des Loges and *Dalopius* Eschscholtz species (Serrano et al. 2022), *Elater abruptus* Say, *M. ignobilis* Melsheimer, *Agriotes insanus* Candèze, *Gambrinus griseus* (Palisot de Beauvois), *G. plebejus* (Say), and *G. rudis* (Brown) (Millar et al. 2024).

Improved knowledge of the sex pheromone communication of pest species enables development of novel IPM tools for their management (Arakaki et al. 2008a, b; Furlan et al. 2021; Kabaluk et al. 2015; Rashed and Wenninger 2023; Vernon and van Herk 2022). Identification of a sex pheromone for *M. communis* (Williams et al. 2019) rapidly translated into applied research on improving trapping technology, and understanding regional phenology and crop type-beetle activity associations (Pellegrino et al. 2021; Schoeppner et al. 2023) that are focused on sustainable solutions to *M. communis* wireworm damage. In addition to *M. communis*, several other *Melanotus* species in North America (e.g., *M. verberans* (LeConte), *M. depressus* (Melsheimer), *M. similis* (Kirby)) are pests of a variety of crops, including maize, small grains, and root/tuber crops (Brown and Keaster 1986; Riley and Keaster 1979; Vernon and van Herk 2022; Ward and Keaster 1977). Discovery of sex pheromones for *Melanotus* pest species will provide new tools for crop protection, as well as expanding our knowledge of pheromone communication in this taxon.

The goal of the present study was to identify, synthesize, and evaluate sex pheromone components of *M. verberans*. This species is found in the eastern U.S. from New York to Florida and west to South Dakota and Texas (Mathison 2021; Quate and Thompson 1967). *Melanotus verberans* is a moderate-sized beetle (9–12 mm length), red-brown and elongate (Mathison 2021; Quate and Thompson 1967). Life history details of this species are poorly known but, like other *Melanotus* species (Douglas et al. 2024), M. *verberans* larvae live in the soil. Depending on local conditions, adult beetles emerge during the spring or early summer to mate, after which females deposit their eggs in or on the soil. After eggs hatch, the larvae (i.e., ‘wireworms’) remain in the soil for several years feeding on plant, and perhaps animal, matter before they pupate (Becker et al. 1991; Vernon and van Herk 2022; Douglas et al. 2024). Duration of the larval period can vary greatly from a few years to nearly a decade (Fenton 1926; Vernon et al. 2009; Vernon and van Herk 2022; Williams unpubl. data). Factors that affect larval duration are poorly understood, but probably include soil and climatic conditions, and food types and availability. Pupation is of 2–3 weeks duration, after which adults emerge and make their way to the soil surface (Fenton 1926). Details of the reproductive physiology of *M. verberans* are scant, but identification of its sex pheromone will make possible basic and applied studies aimed at reducing the species’ pest potential.

## Methods and Materials

### Collection and Maintenance of Beetles

Live *Melanotus verberans* adults were collected from agricultural fields and adjacent wooded areas at the USDA-ARS U.S. Vegetable Laboratory (USVL) in Charleston, SC, USA, (32°44′44.06″N 80°03′40.35″W, elev. 4 m) and Clemson University Pee Dee Research and Extension Center (CUPDREC) in Florence, SC, USA, (34°17′40.60″N 79°43′35.00″W, elev. 35 m) intermittently from April to September in 2023 and 2024. Live beetles were collected for preparation of pheromone extracts at lights (UV and incandescent), by beating vegetation, and by hand. Mating status, age, and other life history factors of the beetles were not known. Additionally, in 2024, male *M. verberans* beetles were live-trapped for coupled gas chromatography-electroantennogram detection (GC-EAD) assays using funnel traps (McQuate 2014) baited with the complete 5-component blend (see below). After collection, beetles were held individually in ventilated transparent plastic vials streaked with honey and containing a piece of paper towel moistened with distilled water. Insects were held (22 °C ± 1, 50% r.h., L: D 16:8) for 1 to 7 d prior to overnight shipment to the University of California, Riverside, Entomology Quarantine Facility (USDA-APHIS permit P526P-20-00853). All *Melanotus* species were identified by comparison with reference specimens of known identity, supported with genital morphology and taxonomic keys (Mathison 2021; Quate and Thompson 1967). Voucher specimens are deposited in the Clemson University Arthropod Collection, Clemson, SC, and the National Museum of Natural History through the USDA-ARS Systematic Entomology Laboratory, Washington, D.C.

### Extraction and Analysis of Potential Sex Pheromone Components

After arrival from shipment, female beetles were cold anesthetized, and the ovipositor and attached glandular structures were pulled out of the abdomen tip with forceps. Ovipositors were extracted individually overnight in 0.1 ml methylene chloride, then the extracts were transferred to screwcap autosampler vials fitted with fused 0.2 ml inserts. Extracts were analyzed by coupled gas chromatography-mass spectrometry (GC-MS) using an Agilent 7820 A gas chromatograph with an autosampler, equipped with an HP-5 column (30 m × 0.25 mm ID × 0.25 µ film thickness, Agilent Technologies, Santa Clara, CA, USA), interfaced to an Agilent 5977E mass selective detector run in electron impact ionization mode (70 eV), with a mass range of *m/z* 40–400. Injections (1 µl) were made in splitless mode (purge valve opened after 30 s). The GC oven was programmed from 40 °C for 1 min, increased 10 °C/min to 280 °C, held for 10 min. Compounds were tentatively identified by interpretation of their mass spectra, and for tetradecyl acetate, a match with a database spectrum (W8N05ST; Wiley version 8.0 and NIST 11, version 5.0). Identifications were confirmed by matching retention times and mass spectra with those of authentic standards.

Antennae from male *M. verberans* beetles live-trapped in 2024 were tested against ovipositor extracts and solutions of authentic standards by GC-EAD analyses. Before male beetles were transfered from the quarantine facility to the GC-EAD laboratory, the legs were removed and their genitalia were glued shut with Super Glue^®^. An antenna was removed from the head with forceps, and mounted between saline-filled capillary glass electrodes, with a gold wire providing connection to a custom-built EAD amplifier. The GC was equipped with an HP-5 column (30 m × 0.32 mm ID × 0.25 µ film thickness, Agilent), using a temperature program as described above. Injections (1 µl) were made in splitless mode. The column effluent was directed into an ‘X’ cross, with half of the sample going to the flame-ionization detector and the other half to the antennal preparation, with the 4th arm of the X-cross providing helium make-up gas. The portion directed to the EAD was diluted with humidified air (~ 550 ml/min), with the flow directed over the antennal preparation. The GC and EAD detector outputs were recorded and displayed with Peak Simple^®^ software.

### Chemicals

Tetradecyl acetate, 13-tetradecenyl acetate, 13-tetradecenyl butyrate, and 13-tetradecenyl hexanoate were synthesized from the respective alcohols and acids or acid chlorides as previously described (Williams et al. 2019; Pellegrino et al. 2021). 13-Tetradecenyl 5-hexenoate was synthesized in analogous fashion from 5-hexenoic acid and 13-tetradecenol with the coupling reagent 1-ethyl-3-(3-dimethylaminopropyl) carbodiimide hydrochloride (EDC) in methylene chloride, with dimethylaminopyridine catalyst. EI mass spectra of all compounds are shown in the online supplement (Figs S1-S5).

### Field Bioassays of Synthetic Pheromone Candidates

In 2023, bioassays were conducted at three locations in South Carolina: USVL, CUPDREC, and a commercial tree farm (CTF) in Liberty, SC (34°46′04.80″N 82°40′21.40″W, elev. 254 m). These field trials were part of a larger study on pheromone discovery in click beetles. Field trials were conducted at three sites (two trials at USVL, and one trial each at CUPDREC and CTF) for 16 wk (16 May – 5 September). One of the USVL transects was at the edge of a woodlot adjacent to a block of organic fields (6 ha), and the other transect was established along an unpaved farm road bisecting a woodlot 1.2 km distant from the first transect. The transect at CUPDREC was established on the edge between a woodlot and a fallow field, and the transect at CTF was established between nursery plots of ornamental trees. Each trap transect was established using Vernon Pitfall Traps^®^ (ca. 17 × 14 cm) (VPT) (van Herk et al. 2018) constructed of black polypropylene and deployed 15 m apart. VPTs were positioned 1 m above the ground on black plastic stakes (Schoeppner et al. 2023). A synthetic pheromone blend approximating the ratio determined from analysis of *M. verberans* ovipositor extracts (100:18:3:40:54, mean ratio from two specimens; 13-tetradecenyl acetate: tetradecyl acetate:13-tetradecenyl butyrate:13- tetradecenyl 5-hexenoate:13-tetradecenyl hexanoate) was used as the test lure. In all trials, trap lures consisted of 9 mm grey rubber septa (West Pharmaceutical Services, Lititz, PA, USA) loaded with the test compounds (4 mg of the major component plus the appropriate amounts of minor components in ratio given above) diluted in 0.1 ml hexane. Solvent control lures were loaded with 0.1 ml of neat hexane. Lures were suspended with a paper clip from the center of the inside of the trap lid. Each VPT held a 250 ml polypropylene sample cup (Falcon 354015, Corning Life Sciences, Corning, NY) containing ca. 12 ml of a 1:1 mixture of water and propylene glycol (Prestone LowTox^®^ Antifreeze/Coolant; Prestone Products Corp., Lake Forest, IL, USA) to serve as a killing agent/preservative. Click beetles were removed from traps weekly and lures were replaced and treatment locations in each transect were re-randomized every 3 wk.

In 2024, field trials were conducted at USVL (three trials) and CUPDREC (two trials). At USVL, the two transects from 2023 were used, and an additional trial was set up on a two-track road bisecting woods. All transects at USVL were within 1.2 km of each other. At CUPDREC, the transect from 2023 was used, and an additional trial was established between a woodlot and a fallow field 1.6 km away from the first transect. Trapping was conducted with the 5-component lure from 16 April – 22 August, except at the new CUPDREC transect (29 May – 22 August). This trapping period was used to characterize the seasonal phenology of beetle activity in 2023 and 2024. Trapping methods were as described for 2023.

Two additional studies were conducted in 2024, which were based on strong responses by the beetles to the complete 5-component blend in 2023. In the first study (16 April – 7 May), we tested the complete 5-component blend against 4-component blends with each one of the 4 components missing. Blends consisted of 1 mg of the major component, 13-tetradecenyl acetate, and appropriate amounts of the minor components, based on the ratio found in ovipositor extracts. This subtractive approach permitted identification of each component’s role in attraction, and demonstrated that 13-tetradecenyl acetate and 13-tetradecenyl hexanoate were necessary bioactive pheromone components. Based on these results, a second blend ratio study (7–28 May) was conducted to better elucidate the role of these two compounds in attraction. A semi-log scale was used, holding the dose of 13-tetradecenyl acetate fixed at 2 mg, and varying the amounts of 13-tetradecenyl hexanoate (from 0 to 2 mg).

### Statistical Analysis

Data analyses were limited to treatments relevant to the target species, *M. verberans*, by omitting treatments that represented pheromones of other elaterid species, none of which caught any *M. verberans* males (data not presented). Replicates were defined by transect-sample date combination. For 2023, numbers of adult male beetles captured were transformed (log_10_(y + 1) to mitigate non-normality (Sokal and Rohlf 1995), and differences between means for treatments and controls were tested using single-factor ANOVA. For the sequential studies in 2024, in each experiment, proportion of male beetles captured in each treatment relative to the total number caught was calculated for each transect-sample date combination, and was arcsine square root transformed. Transformed data for treatments and hexane controls were subjected to single-factor ANOVA followed by (1) Dunnett’s test to compare each treatment to a selected control, i.e., the complete 5-component blend, and (2) Tukey’s HSD for all pairwise comparisons of treatment means. Untransformed mean proportions *±* SE across all sample dates are presented in results. Seasonal phenology is presented as the average number of beetles per trap for each sample date.

## Results

### Identification of Pheromone Candidates

**Fig. 1:**
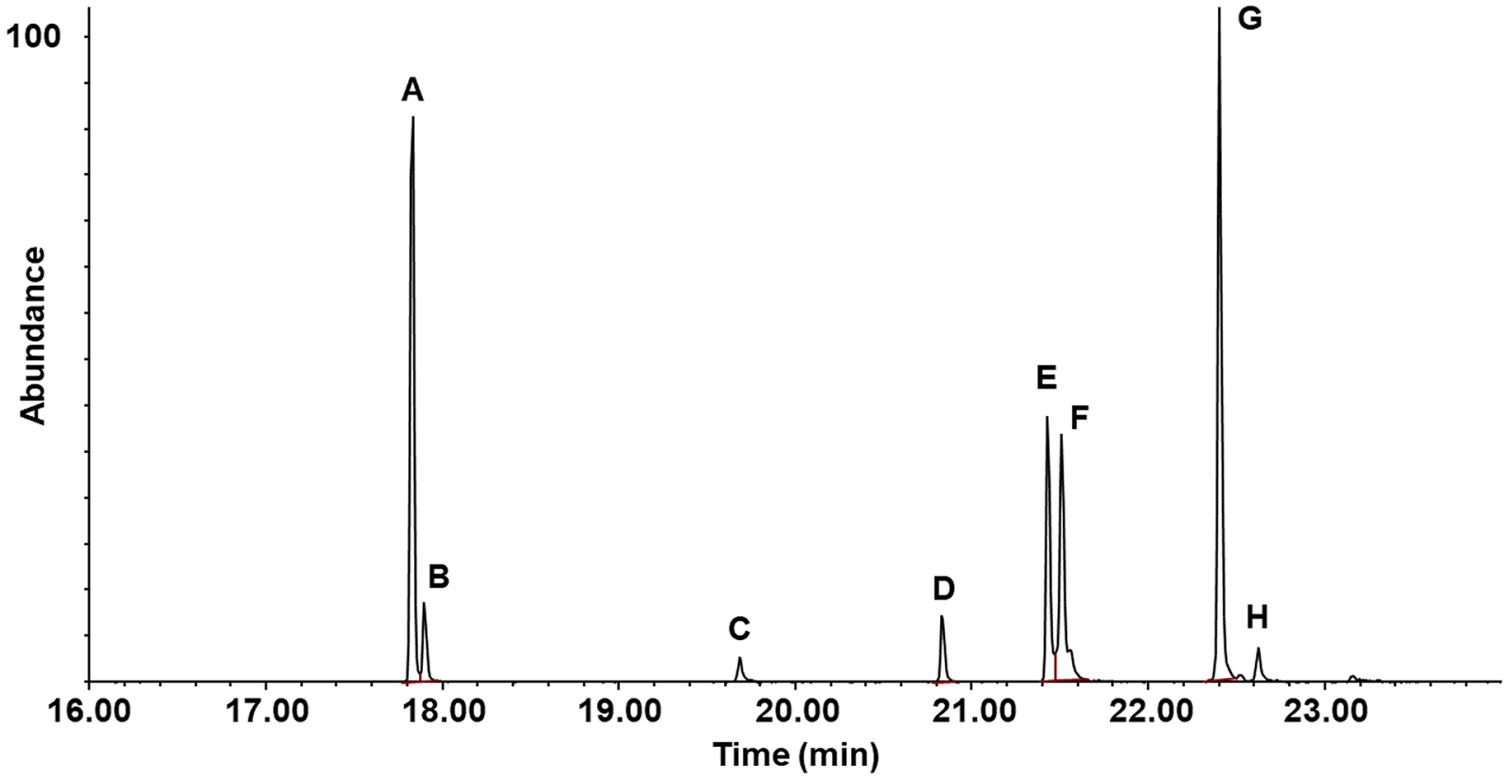
Representative coupled gas chromatography-mass spectrometry trace of an ovipositor extract of a *Melanotus verberans* female. Compound identifications: A, 13-tetradecenyl acetate; B, tetradecyl acetate; C, 13-tetradecenyl butyrate; D, heneicosane; E, 13-tetradecenyl 5-hexenoate; F, 13-tetradecenyl hexanoate; G, tricosene isomer; H, tricosane

Coupled GC-MS analyses of extracts of ovipositors from five individual female *M. verberans* beetles revealed several peaks in the total ion chromatograms (Fig. 1), of which five were assessed to be possible pheromone components, with the remaining peaks being typical straight-chain alkane or alkene insect cuticular hydrocarbons. The five compounds were tentatively identified from interpretation of their mass spectra. Thus, the mass spectrum (Fig. S1) of peak **A** did not have a visible molecular ion, but showed diagnostic peaks at *m/z* 61 (indicative of an acetate ester), and *m/z* 194 (possible C_14_H_26_^+^), suggesting that the other portion of the ester was a C_14_ alcohol with one double bond. This was partially confirmed by the tentative identification of the second peak **B** as tetradecyl acetate, from a database match, and the presence of diagnostic ions at *m/z* 61 (acetate) and *m/z* 196 (C_14_H_28_^+^) (Fig. S2). The molecular ion (*m/z* 256) was not visible. The third peak **C** was tentatively identified as a homolog of the first peak, with *m/z* 194 indicative of a C_14_H_26_^+^ fragment, and prominent ions diagnostic for a butyrate ester at *m/z* 71 and 89 (Fig. S3). The molecular ion (*m/z* 282) was not detected. Peak **D** was readily identified as the cuticular hydrocarbon heneicosane, from its molecular ion at *m/z* 296, and its characteristic straight-chain alkane spectrum. Peak **E** exhibited a small molecular ion at *m/z* 308 (Fig. S4), for a possible molecular formula of C_20_H_36_O_2_, suggesting a 20 carbon ester with two C = C bonds. This was confirmed by the presence of a diagnostic *m/z* 194 ion, indicative of a tetradecenol as the alcohol portion of the ester, and two prominent fragments at *m/z* 97 and 115, indicative of a six carbon, monounsaturated acid as the other piece of the ester. The mass spectrum (Fig. S5) of the fifth potential pheromone candidate, peak **F** suggested that it was an analog of the first and third peaks, with the *m/z* 194 ion indicative of a tetradecenol portion, and prominent diagnostic ions at *m/z* 99 and 117 indicating hexanoic acid as the other portion of the ester. The remaining two peaks **G** and **H** were identified from their mass spectra as a tricosene isomer and tricosane, respectively, from their molecular ions and mass spectra, i.e., two likely cuticular hydrocarbons.

The double bond in the alcohol portion of the compounds with an *m/z* 194 ion was tentatively assigned to the terminal 13 position, based on the fact that terminally unsaturated esters had been identified as pheromones of other *Melanotus* species (*M. sakishimensis*, Tamaki et al. 1990; M. *tamsuyensis*, Yen, 1989; *M. communis*, Williams et al. 2019). In addition, for the 13-tetradecenyl acetate, its Kovats retention index (KI) matched that previously reported for this compound on a DB-5 column, and its KI is distinguishable from those of all other double bond isomers (Marques et al. 2000). For the fourth compound, with an unsaturated six carbon acid portion, the double bond was tentatively assigned to the terminal 5 position. Thus, the identities of the five compounds were tentatively assigned as 13-tetradecenyl acetate, tetradecyl acetate, 13-tetradecenyl butyrate, 13-tetradecenyl 5-hexenoate, and 13-tetradecenyl hexanoate. These identifications were confirmed by matching the retention times and mass spectra of the insect-derived compounds with those of authentic standards.

**Fig. 2:**
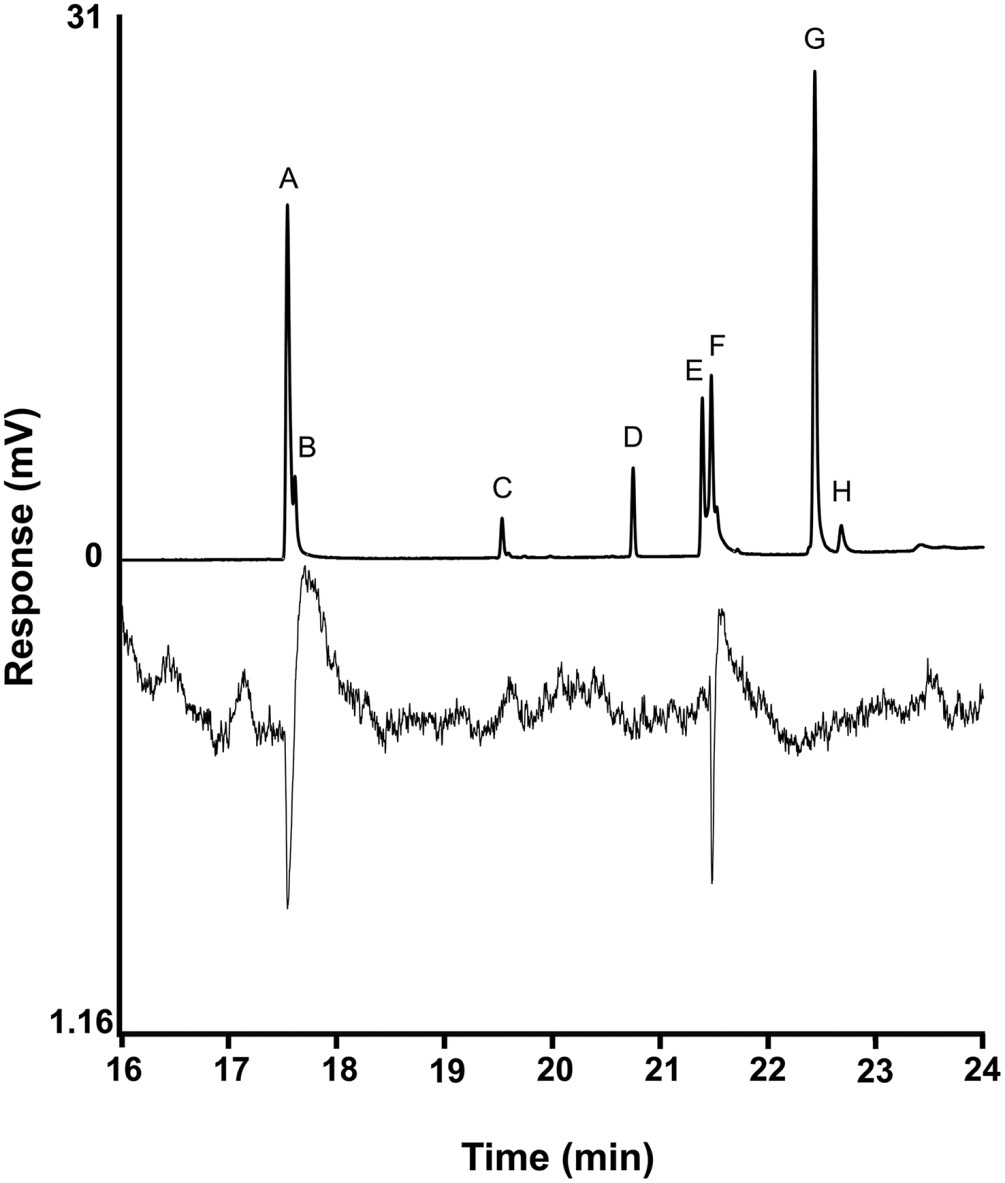
Representative coupled gas chromatography-electroantennogram detection traces of an ovipositor extract of a *Melanotus verberans* female. Upper trace is the gas chromatogram, lower inverted trace is the antennal response of a male beetle. Compound identifications are as in Fig. 1

The extracts were also analyzed by GC-EAD, revealing that only 13-tetradecenyl acetate (peak **A**) and 13-tetradecenyl hexanoate (peak **F**) elicited consistent responses from antennae of male beetles (Fig. 2, S6). These results were confirmed by stimulating antennae with the five synthetic standards. Of the five compounds, 13-tetradecenyl acetate consistently elicited the strongest responses from antennae of males, with smaller responses to 13-tetradecenyl hexanoate, and occasionally, a small response from 13-tetradecenyl 5-hexenoate (Fig. S6). Tetradecyl acetate and 13-tetradecenyl butyrate did not elicit responses from the antennae, either in extracts, or in solutions of standards.

### Field Bioassays of Synthetic Pheromone Candidates

**Fig. 3:**
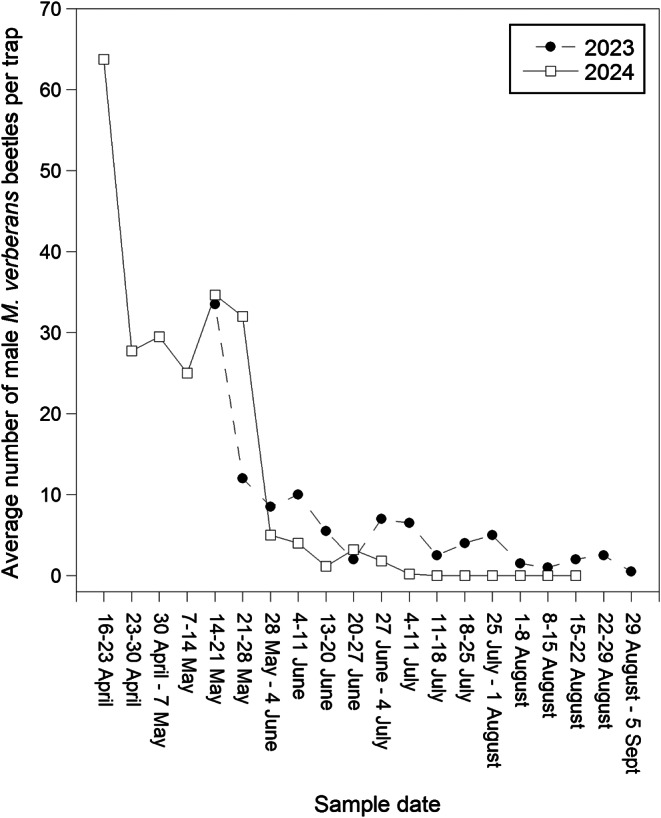
Seasonal phenology of male *M. verberans* captures with the full 5-component blend, 2023 and 2024

In 2023, a total of 263 males were captured in traps baited with the complete blend of the five compounds 13-tetradecenyl acetate, tetradecyl acetate, 13-tetradecenyl butyrate, 13-tetradecenyl 5-hexenoate, and 13-tetradecenyl hexanoate, compared to 0 beetles in control traps (*F* = 276, df = 1,63, *P* < 0.0001). Beetles were captured from 11 May to 5 September 2023 (Fig. 3).

**Fig. 4:**
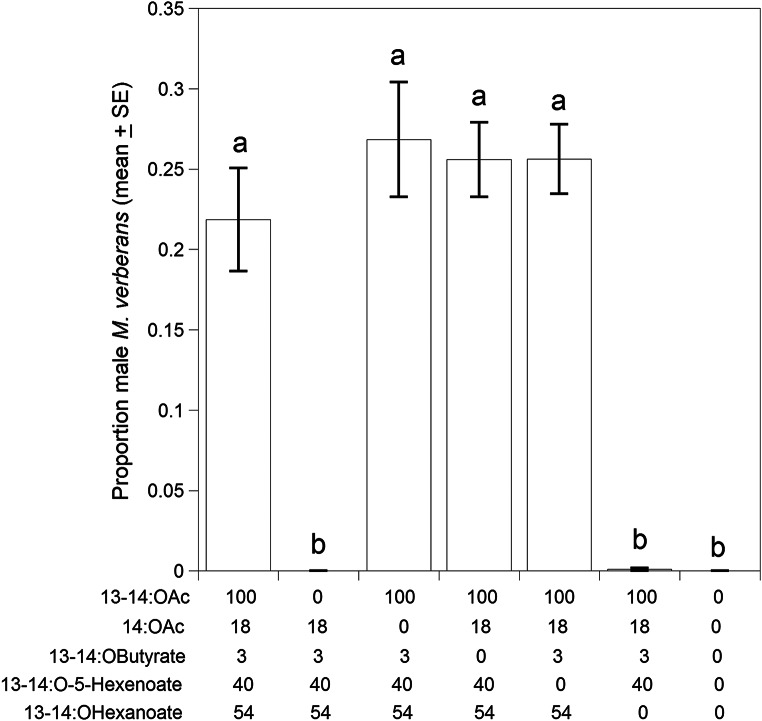
First field bioassay in 2024, testing attraction of male *M. verberans* to the full 5-component blend (column at the left) against 4-component blends in a subtractive design (ratios shown in the X-axis labels). Columns represent the proportion of the total number of male beetles caught for each treatment. Columns surmounted with the same letters are not significantly different (ANOVA, *F* = 80.7, df = 6,76, *P* < 0.0001, *n* = 2009, followed by Dunnett’s Test comparing the 5-component blend to each of the 4-component blends, *P* < 0.05)

The first trial in 2024 tested the full 5-component blend against 4-component blends in a subtractive design, i.e., each treatment consisted of lures with one of the five compounds omitted. A total of 2009 males were captured in traps baited with the treatments compared to 0 beetles in control traps (*F* = 80.7, df = 6,76, *P* < 0.0001) (Fig. 4). Two of the 4-component blends showed significant decreases in captures when compared to the complete 5-component blend; these were the blend without 13-tetradecenyl acetate, where no beetles were captured (|*t*|=11.0, *P* < 0.0001), and the blend without 13-tetradecenyl hexanoate where one beetle was captured (|*t*|=10.8, *P* < 0.0001) (Fig. 4). Captures from the remaining 4-component blends were not significantly different from captures with the complete 5-component blend (*P* > 0.05).

**Fig. 5:**
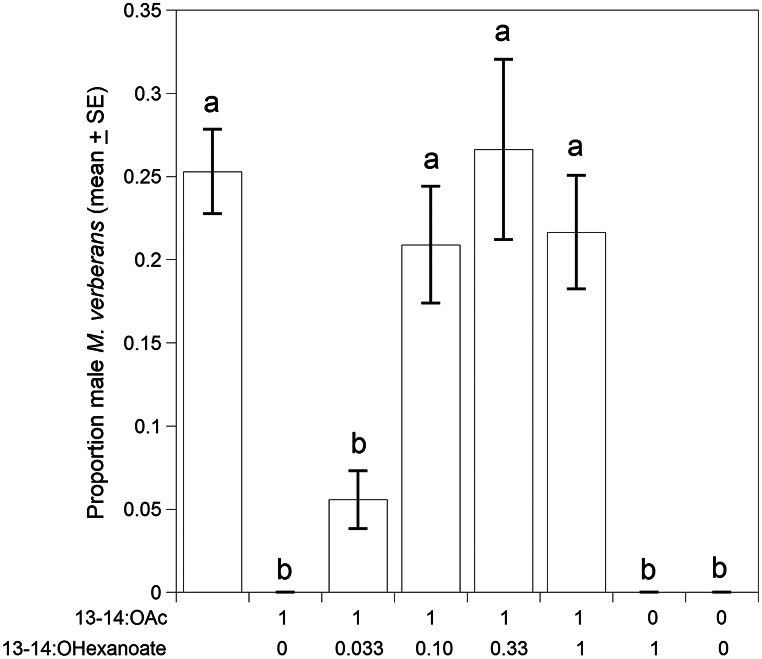
Second field bioassay in 2024, testing attraction of male *M. verberans* to the full 5-component blend (column at the left) and to different ratios (shown in the X-axis labels) of 13-tetradecenyl acetate and 13-tetradecenyl hexanoate. Columns represent the proportion of the total number of male beetles caught for each treatment. Columns surmounted with the same letters are not significantly different (ANOVA, *F* = 47.2, df = 7,71, *P* < 0.0001, *n* = 1166, followed by Tukey’s HSD, *P* < 0.05)

Because the results of the first 2024 trial indicated that 13-tetradecenyl acetate and 13-tetradecenyl hexanoate were both required to obtain strong attraction, a follow-up trial tested different ratios of these compounds. A total of 1166 males were captured in traps baited with the treatments compared to 0 beetles in control traps (*F* = 47.2, df = 7,71, *P* < 0.0001) (Fig. 5). No beetles were captured in treatments where 13-tetradecenyl acetate or 13-tetradecenyl hexanoate were the sole components. The 1:0.033 ratio of 13-tetradecenyl acetate: 13-tetradecenyl hexanoate was more attractive than the control or the individual compounds, resulting in ca. 5% of the total captures in the trial (Fig. 5). However, treatments with greater ratios of 13-tetradecenyl hexanoate in the 2-component blend captured significantly more beetles than the 1:0.033 blend (1:0.10, 21% capture,|*t*|=6.83, *P* < 0.0002; 1:0.33, 27% capture,|*t*|=8.63, *P* < 0.0001; 1:1, 22% capture,|*t*|=7.05, *P* < 0.0001), but were not significantly different from each other or the complete 5-component blend (*P* > 0.05) (Fig. 5). In 2024, beetles were captured from 16 April to 1 August (Fig. 3).

## Discussion

In 2023 we identified five possible pheromone components, including 13-tetradecenyl acetate, tetradecyl acetate, 13-tetradecenyl butyrate, 13-tetradecenyl 5-hexenoate, and 13-tetradecenyl hexanoate, in solvent extracts of genitalia of female *M. verberans*. The full 5-component blend was attractive to male beetles in field trials. However, a subsequent subtractive bioassay in 2024 indicated that only two of the compounds, 13-tetradecenyl acetate and 13-tetradecenyl hexanoate, were both necessary and sufficient for attraction of males. These compounds were only attractive as a blend; each compound alone did not attract any *M. verberans* males. A final bioassay testing different ratios of the two active components showed that the smallest ratio of 13-tetradecenyl hexanoate tested, i.e., 1:0.033 13-tetradecenyl acetate to 13-tetradecenyl hexanoate, was more attractive than controls, but blends with larger ratios of 13-tetradecenyl hexanoate, i.e., 1:0.10 or greater led to increases in attraction similar to the complete 5-component blend. The other three compounds present in female extracts, tetradecyl acetate, 13-tetradecenyl butyrate, and 13-tetradecenyl 5-hexenoate, appeared to be inactive, neither increasing nor decreasing attraction of male *M. verberans*. Our results suggest that the optimal ratio of pheromone components for *M. verberans* lies between the ratios of 1:0.10, 1:0.33 and 1:1 because these binary blends were statistically equivalent, and all were significantly more attractive than the other binary blends tested (1:0, 1:0.033 and 0:1).

Results from electrophysiological trials were consistent with field bioassay data. In GC-EAD assays, only 13-tetradecenyl acetate and 13-tetradecenyl hexanoate elicited strong and reproducible responses from antennae of male beetles, further suggesting that the active attractant consists of only these two components. The possible roles of the other three components remain unclear. They may serve as inhibitory compounds to minimize cross-attraction of congeners that might also use 13-tetradecenyl acetate and/or 13-tetradecenyl hexanoate as pheromone components. Alternatively, they may be artifacts of the way the extracts were prepared; we analyzed extracts of ovipositors, because all our attempts to collect the volatiles actually released by calling females of *Melanotus* species have been unsuccessful (Williams et al. 2019). It is possible that a compound like tetradecyl acetate might be present as a biosynthetic intermediate, but it is more difficult to make a case that 13-tetradecenyl butyrate or 13-tetradecenyl 5-hexenoate might be biosynthetic precursors of 13-tetradecenyl acetate or hexanoate.

This phenomenon of click beetle females producing analogs and homologs of the compounds that constitute their actual sex pheromone blend appears to be widespread within the Elateridae. For example, Tolasch et al. (2007) found that pheromone gland extracts of *Elater ferrugineus* L. contained 7-methyloctyl 5-methylhexanoate, 7-methyloctyl octanoate, 7-methyloctyl 7-methyloctanoate, and 7-methyloctyl (*Z*)-4-decenoate, and the full blend of compounds was attractive. However, a follow-up study showed that only the latter compound was required to get full activity (Svensson et al. 2012). In another genus, *Dalopius marginatus* (L.) gland extracts contained a major component (neryl decanoate) and two homologs, neryl octanoate and neryl dodecanoate, but neryl decanoate alone was sufficient for attraction (Tolasch and Steidle 2022). Similarly, volatiles collected from the crushed abdomens of *Idolus californicus* Desbrochers des Loges contained neryl hexanoate and neryl octanoate, but neryl hexanoate was the sole attractive component (Serrano et al. 2022). In the genus *Agriotes* Eschscholtz, both gland extracts and volatiles collected from female *A. sordidus* Illiger contained two major components, geranyl hexanoate and farnesyl hexanoate, but geranyl hexanoate was fully active as a single component, with blends being no more attractive. Another example would include *Parallelostethus attenuatus* (Say), where volatiles collected from crushed abdomens of females contained hexanoic acid, 1-octanol, 1,8-octanediol, octyl hexanoate, 1,8-octanediol monohexanoate, and 1,8-octanediol dihexanoate, with the latter compound being fully attractive, and blends with other components actually being less attractive (Millar et al. 2022). It is unclear why these species are producing what are apparently redundant or even inhibitory compounds, which complicate the process of trying to sort out what constitutes the active pheromone blend for each species. Additional examples of elaterid pheromone gland extracts containing complex mixtures of analogs and homologs are listed in Tóth (2013). Given so many cases across multiple genera, additional examples will undoubtedly emerge as pheromone blends are identified for new species.

During our field trials we observed no evidence of cross-attraction of other elaterid species to any of the lures tested, despite numerous elaterid species being captured at our field sites using other collecting methods. Thus, our data indicate that the blend of 13-tetradecenyl acetate and 13-tetradecenyl hexanoate may be species-specific to *M. verberans*. These results suggest that the pheromone components or pheromone blends of *Melanotus* and other click beetle species may be both diverse, but also narrowly tuned, with each species using either relatively unique compounds, or species-specific ratios of compounds that may be shared by congeners. In both years, flight activity of *M. verberans* males was greatest in April through May, after which captures were greatly diminished. This peak of flight activity is relatively early in the season, and also may play a role in promoting reproductive isolation between congeners that share pheromone components.

Sex pheromones or attractants have been identified for three other North American *Melanotus* species. The major sex pheromone component for *M. communis* has been identified as 13-tetradecenyl acetate (Williams et al. 2019), whereas the congeneric *M. ignobilis* is strongly attracted to 11-dodecenyl butyrate, suggesting that this compound is a likely pheromone component for this species (Millar et al. 2024). Several decades ago, *Melanotus depressus* (Melsheimer), but not *M. verberans*, was reported to be attracted to the tufted apple bud moth (*Platynota idaeusalis* Walker) sex pheromone (2:1 ratio of (*E*)-11-tetradecenyl acetate + (*E*)-11-tetradecen-1-ol) (Brown and Keaster 1983). Like *M. verberans*, *M. communis* females also produce tetradecyl acetate, and in both species it appears to be inactive. The strong species-specific attraction of the blend of 13-tetradecenyl acetate and 13-tetradecenyl hexanoate produced by *M. verberans* appears to be a crucial factor in maintaining effective reproductive isolation between it and *M. communis*, because no *M. communis* were caught in traps baited with the blend despite both species sharing 13-tetradecenyl acetate. As described above, the early-season activity of the *M. verberans* beetles may also play a role in reproductive isolation between congeners.

Pheromones have been reported for two other *Melanotus* species, both from Japan. Thus, *Melanotus okinawensis* Ôhira uses the single component dodecyl acetate (Iwanaga and Kawamura 2000; Tamaki et al. 1986), whereas a blend of (*E*)-9,11-dodecadienyl butanoate and (*E*)-9,11-dodecadienyl hexanoate are used by *M. sakishimensis* Ôhira (Iwanaga and Kawamura 2000; Tamaki et al. 1990). The similarities of these compounds to those produced by their North American congeners are obvious. Related compounds that may be attractive have been identified in other *Melanotus* species, although bioassay data are lacking. For example, as for *M. sakishimensis*, (*E*)-9,11-dodecadienyl butanoate and (*E*)-9,11-dodecadienyl hexanoate have been identified in extracts of *M. tamsuyensis* Bates (Yen and Chen 1998). The European species *M. punctolineatus* (Pelerin) may use a tetradecenyl butyrate as a pheromone, whereas *M. rufipes* (Herbst) may use a tetradecadienyl butyrate (Tolasch et al. 2007). Pheromone glands of the west-Eurasian *M. fusciceps* (Gyllenhal) and the Holarctic *M. castanipes* (Paykull) contained mixtures of > 22 compounds, which included esters of 12-carbon and 14-carbon saturated and unsaturated alcohols, although the exact structures were not identified (Yatsynin et al. 1996). In sum, this fragmentary data indicates that there is substantial conservation of general structural types in pheromone components within the *Melanotus* genus, but it is also clear that pheromone blends are likely to be species-specific because of the demonstrated lack of attraction of congeners in the field trials described here and in the other studies described above.

Insect sex pheromones have been integral components of pest management for more than 50 years. More recently, the pheromones of click beetles have been utilized in studies aimed at a better understanding of click beetle biology with emphasis on improving pest control. For example, in North America and Europe, pheromone-based methods have been particularly valuable for monitoring pest species (e.g. Blackshaw and Vernon 2006; Kudryavtsev et al. 1993; van Herk et al. 2021b; Vernon and Tóth 2007), as well as facilitating studies of non-pest species (Larsson 2016; Millar et al. 2022). Such methods also have been used to monitor several invasive Eurasian click beetle species, i.e., *Agriotes* spp., that are now established in North America (Vernon et al. 2001; Vernon and van Herk 2022). In addition, sex pheromones have been used in management of pest species, including mass trapping (Arakaki et al. 2008b; Vernon et al. 2014), and mating disruption of *M. okinawensis* (Arakaki et al. 2008a), and may have potential in attract-and-kill strategies that combine attractants with entomopathogens (Kabaluk et al. 2015). Recently, the *M. communis* pheromone has been used to optimize trapping technology and characterize regional phenology and crop type-beetle activity associations (Pellegrino et al. 2021; Schoeppner et al. 2023). The identification of pheromones and sex attractants for additional species will provide new tools for developing a better understanding of elaterid biology, and management of pest species (Rashed and Wenninger 2023; Rashed and van Herk 2024; Vernon and van Herk 2022).

## Electronic Supplementary Material

Below is the link to the electronic supplementary material.

Supplementary Material 1

Supplementary Material 2

## Data Availability

The authors declare that the data supporting the findings of this study are available within the paper and its Supplementary Information files. Should any raw data files be needed in another format they are available from the corresponding author upon reasonable request.
