# Supplementary material for: Discovery and Field Evaluation of Sex Pheromone Components for the Click Beetle Melanotus verberans (LeConte) (Coleoptera: Elateridae)
Source: J Chem Ecol. 2025 Feb 5;51(1):24. doi: 10.1007/s10886-025-01569-3 (PMC11799071; doi:10.1007/s10886-025-01569-3)
Supplement: Supplementary file 1 — Supplementary Material 1 [file 10886_2025_1569_MOESM1_ESM.docx]

**Supplementary Online Information**

**Discovery and field evaluation of sex pheromone components for** **the click beetle *Melanotus verberans* (LeConte) (Coleoptera: Elateridae)**

**Livy Williams III^1*^· Sean T. Halloran^2^ · Paul D. Baker^3^ · Frank E. Etzler^4^ · Lance L. Lawrence^1^ · Jocelyn G. Millar^2^**

^1^USDA-ARS U.S. Vegetable Laboratory, 2700 Savannah Highway, Charleston, SC 29414, USA. ([livy.williams@usda.gov](mailto:livy.williams@usda.gov)) Orcid: 0000-0003-3179-5232 (Williams); (lance.lawrence@usda.gov)

^2^Departments of Entomology and Chemistry, University of California, Riverside, CA 92521, USA. ([millar@ucr.edu](mailto:millar@ucr.edu)) Orcid: 0000-0001-7639-9001 (Millar); (sean.halloran@ucr.edu) Orcid: 0000-0001-5445-310X (Halloran)

^3^Department of Entomology, Clemson University Pee Dee Research and Education Center, 2200 E. Pocket Road, Florence, SC 29506, USA. ([pdbaker@clemson.edu](mailto:pdbaker@clemson.edu)) Orcid: 0000-0002-3394-6850

^4^Montana Department of Agriculture, 302 N. Roberts, Helena, MT 59714, USA. ([frank.etzler@mt.gov](mailto:frank.etzler@mt.gov)) Orcid: 0000-0001-8192-4667

Figure S1. EI mass spectrum of 13-tetradecenyl acetate

Figure S2. EI mass spectrum of tetradecyl acetate

Figure S3. EI mass spectrum of 13-tetradecenyl butyrate

Figure S4. EI mass spectrum of 13-tetradecenyl 5-hexenoate

Figure S5. EI mass spectrum of 13-tetradecenyl hexanoate


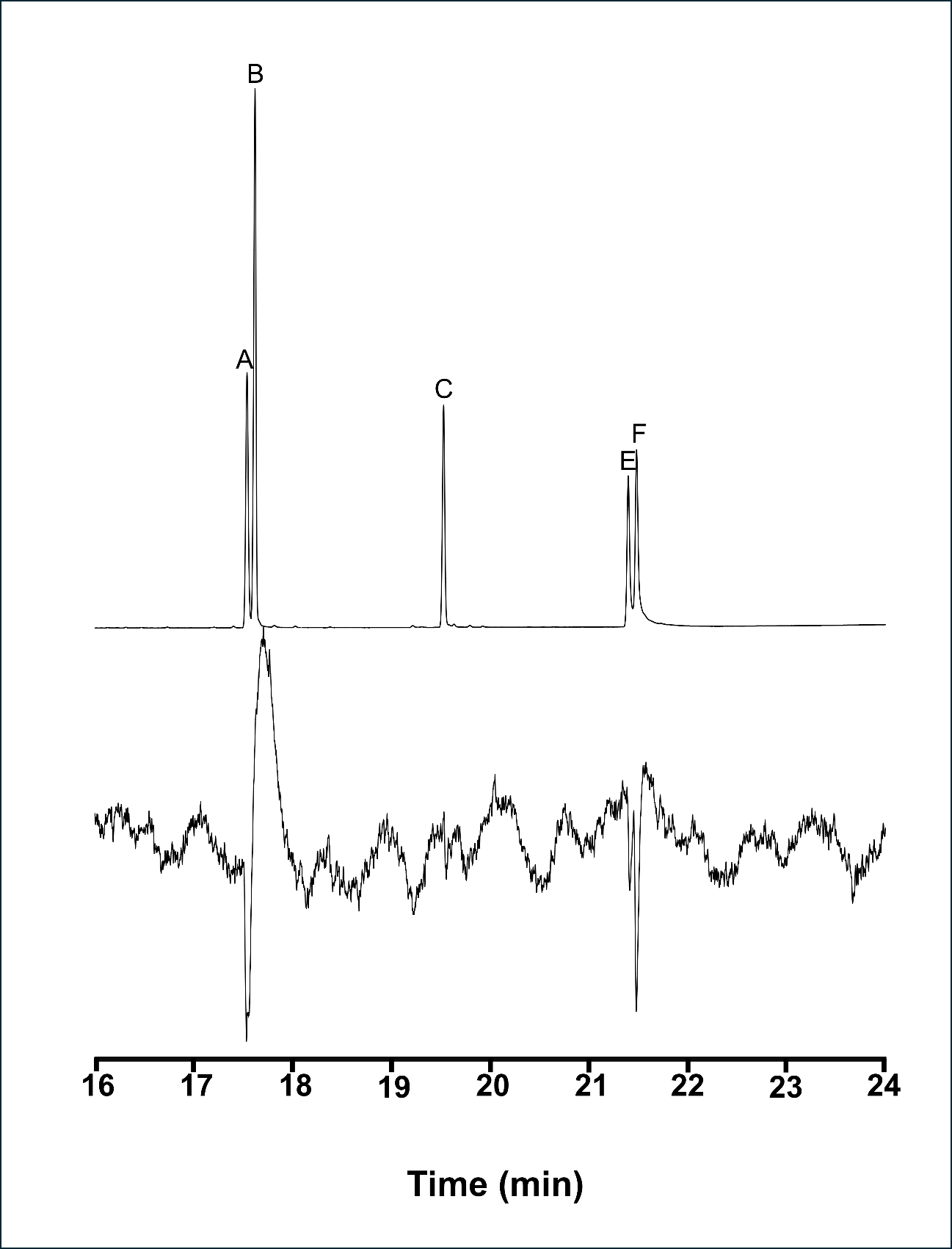


Figure S6. Representative coupled gas chromatography-electroantennogram detection traces of responses of an antenna from a *Melanotus verberans* to synthetic standards. Upper trace is the gas chromatogram, lower trace is the antennal response of a male beetle. Compound identifications: A, 13-tetradecenyl acetate; B, tetradecyl acetate; C, 13-tetradecenyl butyrate; E, 13-tetradecenyl 5-hexenoate; F, 13-tetradecenyl hexanoate.
